# Increasing pro-environmental behavior in the home and work contexts through cognitive dissonance and autonomy

**DOI:** 10.3389/fpsyg.2023.1199363

**Published:** 2023-06-07

**Authors:** Dominik Bentler, Gizem Kadi, Günter W. Maier

**Affiliations:** Department of Psychology, Industrial and Organizational Psychology Laboratory, Bielefeld University, Bielefeld, Germany

**Keywords:** cognitive dissonance, pro-environmental behavior, employee pro-environmental behavior, pro-environmental attitudes, autonomy

## Abstract

The aim of this study was to develop a novel cognitive dissonance intervention founded on the action-based model for enhancing pro-environmental behavior. Based on intraindividual feedback on the expression of personal pro-environmental attitudes and behavior the study confirms the effect of cognitive dissonance intervention to foster pro-environmental behavior. The effect of this intervention could be demonstrated for the home as well as for the work context, although the effects for the work domain were lower. This can be explained by specific situational conditions of the work domain. Autonomy for pro-environmental behavior is significantly lower in the work context than in the home context and significantly moderates the effect of the cognitive dissonance intervention. The present work provides information on how pro-environmental behavior can be influenced in different contexts as well as the significance of situational framework conditions for the effect of behavior-changing interventions.

## 1. Introduction

The goal of the Paris Climate Agreement to keep the temperature from rising more than two degrees Celsius compared to the pre-industrial age poses an extreme challenge for the 196 nations involved in the agreement (Paris Agreement, 2015). The development and application of special technologies, for example e-and hydrogen mobility, the use of renewable energy sources and green IT contribute to achieving these goals. Regardless of these technological developments, the achievement of climate goals depends also significantly on decisions and human behavior. After all, people’s behavior contributes massively to the amount of CO2 released (Gardner and Stern, 2008). Yet, especially in comparison to the home context, the working context is of great interest regarding environmentally beneficial behavioral changes. In Germany, for example, companies are responsible for about 66% of CO2 emissions (Statistisches Bundesamt, 2014). The 3,000 largest companies worldwide cause environmental damage amounting to approximately $2.15 trillion annually (Principles for Responsible Investment, and UNEP Finance Initiative, 2011). The behavior of employees has a significant influence on the environmental performance of a company (Ramus and Steger, 2000). In order to change and increase the environmentally friendly behavior of people, a variety of intervention methods have already been investigated in the home context (Osbaldiston and Schott, 2012), but not in the work context so far. The use of methods for stimulating cognitive dissonance represents the procedure with the highest effect sizes (Hedges’ *g* 0.93) to foster pro-environmental behavior (Cook and Berrenberg, 1981; Steg and Vlek, 2009; Osbaldiston and Schott, 2012). Cognitive dissonance theory focuses on situations in which individuals perceive cognitions, attitudes, or behaviors that are inconsistently expressed and result in a state of arousal. Motivation arises to resolve this arousal. The inconsistency between cognitions, attitudes, and behavior is reduced by changing one of these constructs to achieve relaxation (Harmon-Jones and Mills, 2019). Compared to the results of cognitive dissonance intervention methods, other procedures have smaller effect sizes. Goal setting methods are effective with Hedges’ *g* 0.69 and social models have an effectiveness with *g* 0.63 (Osbaldiston and Schott, 2012). The advantage of cognitive dissonance interventions over goal-setting methods (e.g., Dwyer et al., 1993; Abrahamse et al., 2005) and social models (e.g., Lehman and Geller, 2004; Kazdin, 2009) lies not only in their higher effect sizes but also in their efficient application and implementation. Cognitive dissonance methods are suitable for generating motivation for a behavioral change in a short amount of time without being dependent on other persons. Given these results, it is surprising as to why intervention methods for increasing environmentally sustainable behavior, which have been established in the home context, have found little application in the work context. Therefore, there is a need to identify reasons, if situation-specific conditions exist, for why these intervention methods from the home context are not applied in the work sector, although the environmental impact of industry is significantly higher. In fact, the method of inducing cognitive dissonance has a high impact on changing environmental behavior in the home context but has not even been transferred to change employee’s habits in the working context (Lo et al., 2012; Young et al., 2015).

Research on environmentally friendly behavior aims to contribute to advancing the achievement of the goals of the Paris Climate Agreement (Paris Agreement, 2015). Consequently, in addition to antecedents for pro-environmental behavior, intervention methods for behavioral changes are discussed and tested in current research. The present study first answers the research question why so far only intervention methods in the home sphere have been investigated to foster pro-environmental behavior in comparison to the work context. For this reason, a novel intervention method for increasing sustainable behaviors in the home and work context is developed, which proves that environmentally friendly behavior can also be increased in the work context. Finally, the question is answered which situational conditions in the home and work context influence environmentally friendly behavior. In addition to diagnosing these situational conditions, the specific influence of autonomy is examined in order to be able to develop strategies in future research on how these frameworks can be realigned so that individuals can easily show pro-environmental behavior. This study extends the current state of research by first developing a novel cognitive dissonance method that links intraindividual pro-environmental attitude and behavior data to generate cognitive dissonance and increase future pro-environmental behavior. Compared to existing cognitive dissonance methods, the method developed here stands out for its simplicity in generating an arousal state and thus promises an easy transfer to several application fields. Second, this study is the first investigation to diagnose situational conditions, why the effect of green intervention methods differ in the home and the work context. Third, the effect of these situational frameworks is examined specifically for the home and work contexts to determine whether they have a greater impact on the extent of green behavior in the home or work context, providing insights for the development of further intervention methods. Thus, the results of this study provide significant added value for the use of intervention methods to increase pro-environmental behavior both for the field of environmental research and for implementation in practice. For the field of environmental research, the results display the importance of situational frameworks in the implementation of intervention methods and encourage to diagnose further situational factors that may influence the enhancement of pro-environmental behavior. In addition, the research offers a high potential for application in home as well as work environments and can support individuals, groups and whole organizations in achieving their environmental goals and thus contribute to the achievement of the goals of the Paris Climate Agreement.

## 2. Theoretical background

### 2.1. Literature review

#### 2.1.1. Pro-environmental attitudes and pro-environmental behavior

Since the first and most prominent scale to measure ecological attitudes and knowledge was published by Maloney and Ward (1973), a rising number of studies in the field of environmental psychological research followed (Tian and Liu, 2022). Due to the increasing number of research results in this field, an attempt was made to structure pro-environmental behavior through a framework (Stern, 2000). The basis for this is the division into the public, home and other pro-environmental behavior. In the public sphere, a further distinction is made in terms of the degree of proactivity. If someone wants to actively participate in movements or organizations, it stands for so-called environmental activism, whereas nonactivist behavior describes support in a subtle way, e.g., acceptance of environmentally friendly guidelines (Stern, 2000). In addition, pro-environmental behavior is studied in the home sphere (e.g., recycling behavior or energy conservation behavior; Bamberg and Möser, 2007). This pro-environmental behavior is often described in terms of very concrete behavior and thus differs in its degree of abstraction from public pro-environmental behavior.

Even if most of this research focused on environmental sustainability in the home context, since the early 2000s, there has been an increase in research concentrated on the work context (Young et al., 2015). The majority of environmental sustainability research has focused on pro-environmental attitudes (PEA) and pro-environmental behavior (PEB) in the home and work contexts. PEA can be defined as one’s tendency to exhibit favor toward the natural environment (Hawcroft and Milfont, 2010). PEB describes all individual behaviors that contribute to environmental protection or reduce environmental damage (e.g., energy consumption, waste avoidance, recycling, sustainable consumer and travel behavior; Steg and Vlek, 2009). The relationship of PEA and PEB is most often explained through the Theory of Planned Behavior (TPB; Bamberg and Möser, 2007; Yuriev et al., 2020). TPB assumes that behavior is determined by social norms, perceived behavioral control and personal attitudes (Ajzen, 1991). The evidence confirms that, of these three factors, especially PEA is most strongly related to PEB (Bamberg and Möser, 2007). These findings have been applied to both the home and the work context (Greaves et al., 2013; Judge et al., 2019) and promise excellent potential for using this correlation from PEA and PEB for intervention methods to increase sustainable behaviors.

In the home context, the extent of these behavior is mostly individually controlled, even if structural and economic conditions (e.g., the availability of public transportation) may encourage or inhibit this behavior (Ones et al., 2015). According to TPB studies in the home context, PEA is closely related to PEB (Kaiser and Schultz, 2009). However, the direction of action of the relationship between the two constructs is not yet clear (Prati et al., 2017). While the majority of current studies assume that PEA influences PEB (Hansmann and Binder, 2020), there are also studies that assume the opposite direction of action (Ertz and Sarigöllü, 2019). It is indisputable that PEA and PEB are associated with each other, but the interaction between attitudes and behavior depends on the context. Whereas PEA can predict PEB in the home context, there are no significant correlations between PEA in the home context and PEA or PEB in the work sphere (Manika et al., 2015). Thus, there must be situational factors in the work context that significantly inhibit the relation between PEA and PEB during work and do not exist in the home context.

In comparison to PEB in the home sphere, employee-PEB is limited to any kind of PEB related to the work context (Ones et al., 2015) and can be classified by three dimensions (Zacher et al., 2023). First, PEB can be conducted during work within or outside the employee’s role/work activity. Second, employees can use PEB to have a direct impact on the environment (e.g., employees act to benefit or harm the environment themselves) or indirectly (e.g., employees try to convince their colleagues, supervisors, or others to behave in a more environmentally friendly way). Third, PEB can be low intensity (e.g., saving energy) or high intensity (e.g., designing proposals for sustainable organizational development).

As described, PEB during work can be directly related to the work activity. Compared to PEB in a public or home sphere is not in the type of the behavior that differs to PEB in the work context, but in particular through further (required work) behavior that is associated with it and thus significantly influences the extent of PEB during work. In contrast to PEB in the public and home contexts, PEB in the work sphere is often not determined or controlled by the employee, but rather largely determined by the work activity itself or given organizational guidelines (Ones and Dilchert, 2012). Similar effects by contextual influences on PEB have already been demonstrated in other domains, such as consumer behavior (Ertz et al., 2016). Even though PEB in the home sphere can be restricted by structural conditions, these restrictions can be much more extensive in the work context (Littleford et al., 2014). Studies have shown that PEB at work is significantly influenced by the available autonomy during work activities (Tian et al., 2020).

#### 2.1.2. The role of autonomy for pro-environmental behavior

Many different situational and contextual factors that can promote or inhibit PEB are discussed (Steg and Vlek, 2009). In comparison with the home sphere, behavior-regulating factors are more present in the work sphere. For this reason, situational factors relating to PEB have increasingly been studied in the workplace (Bamberg and Möser, 2007; McDonald, 2014). Primarily, individual behavior at work is determined by the task itself, with the goal of achieving the required work performance. Since organizations as economic enterprises are interested in the efficient achievement of goals, the behavior of the employees with regard to the work activity is controlled to a large extent by work design as a choice of the organization (Hackman and Oldham, 1974; Humphrey et al., 2007). Accordingly, the autonomy of behavior is significantly restricted by the work designed by the organization. Employees are regulated in their behavior in such a way that they do not feel they have the opportunity and the locus of control to show a high degree of employee PEB (Hansmann et al., 2020; Reegård and Drøivoldsmo, 2020). Yuriev et al. (2018) pointed out that the lack of autonomy is one of the main barriers in the organizational context that hinders PEB. Furthermore, studies have confirmed that perceived behavioral control is important to show PEB in the home context (Bamberg and Möser, 2007) as well as in the work context (McDonald, 2014). For this reason, the role of autonomy should be considered in studies to examine the effects of autonomy on the impact of interventions for each context. If interventions are developed to increase PEB, specific factors of the contexts must be considered in order to be able to achieve the highest possible effect of the respective intervention method. Even though there have been studies on interventions to foster PEB in the home context, there is a lack of research into the impact of PEB interventions in the work context. Studies have been able to demonstrate that PEB in both the home context (Gardner and Stern, 2008; Arnold et al., 2018) and the work context (Scherbaum et al., 2008; Staddon et al., 2016) is positively associated to the climate and thus contributes to achieving the goals of the Paris Climate Agreement. It should be considered that autonomy can shed light on why simply transferring intervention methods from the home to the work setting to foster PEB does not work.

### 2.2. Theoretical framework

#### 2.2.1. Cognitive dissonance research

**Figure 1:**
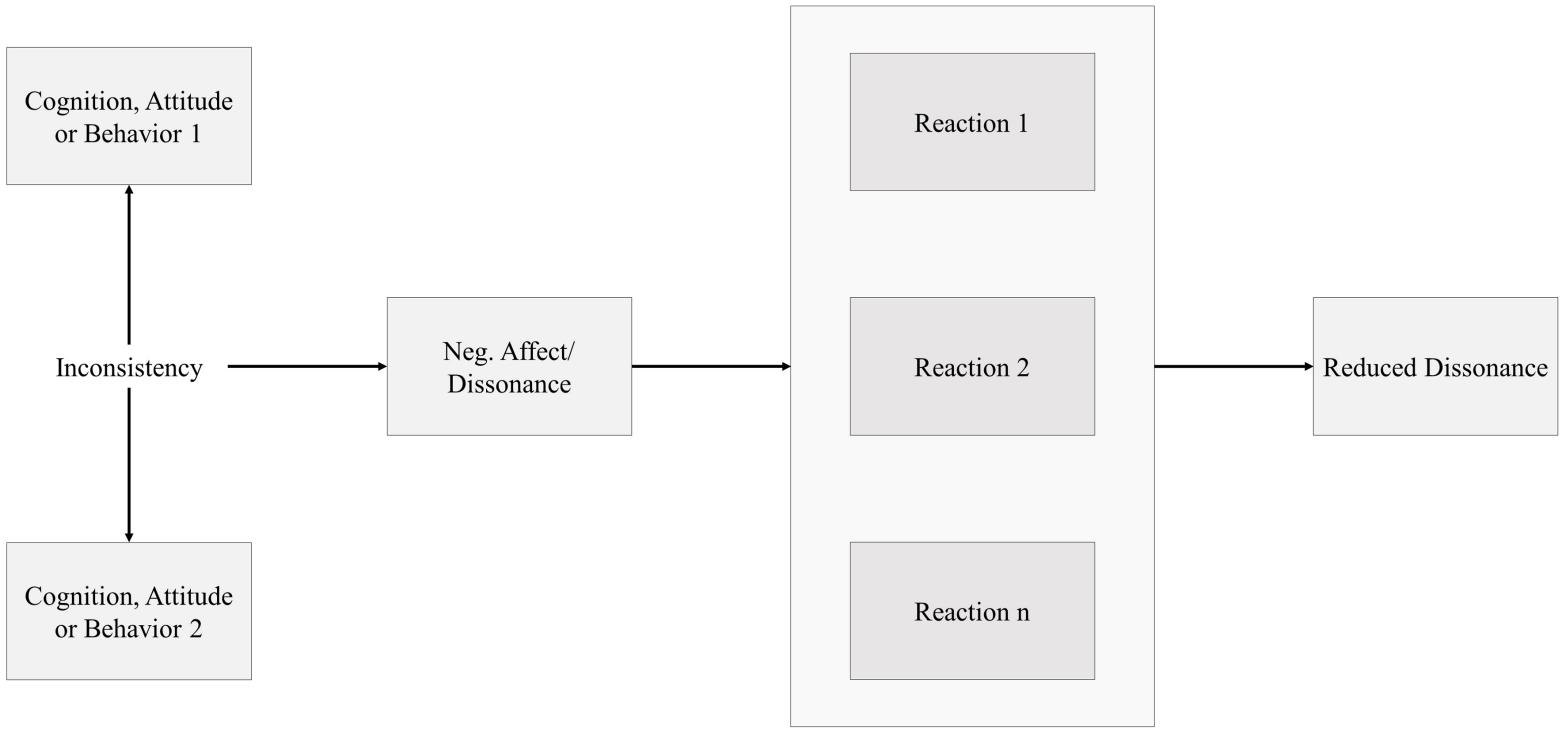
The action-based model (*cf.* Harmon-Jones et al., 2015).

Since cognitive dissonance interventions were invented by Festinger (1957), they have been applied in an enormous number of scientific studies in social and organizational psychology (Hinojosa et al., 2017; McGrath, 2017; Priolo et al., 2019). Cognitive dissonance theory assumes that humans strive for consistency and therefore actively try to reduce cognitive dissonance (Festinger, 1957). Dissonance between the cognitions, attitudes and behaviors that a person experiences through self-perception is thought to produce a motivation that results in genuine cognitive changes (Bem, 1967; Brehm, 2007). Current research assumes that the origin of dissonance and the choice of the way to reduce dissonance can be explained by the action-based model (Harmon-Jones et al., 2015; Harmon-Jones and Mills, 2019). The action-based model assumes that the self-perception and intraindividual comparison of cognitions, attitudes and behaviors have implications for the way people behave. If these cognitions, attitudes and behaviors are inconsistent, this leads to an arousal. This arousal leads to a negative affective reaction, which triggers perceiving dissonance. This dissonance then motivates the subject to reduce the negative affect by changing cognitions, attitudes or behaviors (Harmon-Jones et al., 2009). Of course, a behavioral change as a reaction on this arousal can only take place if the person has sufficient autonomy to do so. The main advantage of the action-based model is especially the simplicity of the theory in comparison to alternative models of cognitive dissonance research. This simple structure of the action-based model simplifies the application of this cognitive dissonance intervention into different contexts. In addition, the assumption of the action-based model has been confirmed in numerous behavioral and neuroscientific experiments (Hajcak and Foti, 2008; van Veen et al., 2009; Mengarelli et al., 2015; Leeuwis et al., 2022). The basic structure of the action-based model is shown in Figure 1.

While the original version of cognitive dissonance theory assumed that, in order to reduce tension, only the attitudes of individuals can be modified (Festinger, 1957), more recent studies have shown that also behavioral changes are used for stress relief (Dickersion et al., 1992; Fointiat, 2004). A change in behavioral habits is not the most convenient way for people to reduce dissonance, but Festinger (1999) describes a change in behavior as the major avenue for cognitive dissonance theory. The basic prerequisite for this behavioral change is, of course, a sufficient degree of autonomy.

#### 2.2.2. Cognitive dissonance interventions

In order to be able to actively control changes initiated by cognitive dissonance, intervention methods have been developed and applied in various situations, e.g., leader-member exchange (Erdogan et al., 2004), prejudice reduction (Heitland and Bohner, 2010), health behavior (Freijy and Kothe, 2013) or nutritional behavior (Bouwman et al., 2022). In the sense of the action-based model, an interaction of two unequal cognitions, attitudes and/or behaviors causes a state of arousal if one becomes aware of the inconsistency between the constructs and, according to the situational conditions and individual abilities, this state of arousal leads to a change in one of these constructs. While behavioral changes are often perceived as a demanding task, such behavioral adjustments are still chosen when the dissonance is high (Leippe and Eisenstadt, 1999). Furthermore, our everyday behavior is characterized by habituation. Changing these habits requires a high degree of attention and effort. Changes in habituated behavior could be postponed by adjusting the underlying cognitions or distract from the dissonance to reduce the arousal (Neal et al., 2006). Although intervention methods related to cognitive dissonance theory are well-researched, this has not been adequately applied in the context of PEB. Studies have shown that especially in the field of PEB, behavioral change through cognitive dissonance is difficult to achieve (Bosone et al., 2022). Even if the application of the theory in the home context is promising, application in the work context presents extensive challenges due to the contextual conditions presented above. Obviously, the goals of the Paris Climate Convention Agreement can only be achieved if people behave in a more environmentally friendly way. Studies have shown that the discrepancy between PEA and PEB that is necessary to induce cognitive dissonance does actually exist (Franzen and Vogl, 2013; Lin et al., 2018). According to the action-based model, this discrepancy between PEA and PEB provides an optimal basis for inducing an arousal in humans.

### 2.3. Conceptual framework

While Osbaldiston and Schott (2012) found 87 publications containing 253 experimental treatments that measured and observed PEB in daily life, Lo et al. (2012) investigated only ten intervention studies to increase PEB in the work context. This gap can be explained by two reasons. Firstly, environmental-psychological studies in the home context have been conducted since the 1970s, whereas research in the work context has only emerged since the millennium (Young et al., 2015). Secondly, and more importantly, due to the situational complexity in the work context described above, the implementation of intervention studies in the work context is more difficult than in the home context (McDonald, 2014). However, based on higher CO2 emissions compared to the home context, the work context represents a research field with a high degree of effectiveness in achieving climate goals. Moreover, while Osbaldiston and Schott (2012) diagnosed cognitive dissonance theory as the most powerful intervention method to foster PEB in the home context, there is no evidence that cognitive dissonance interventions function in the work context as well. Furthermore, there are no findings on the use of cognitive dissonance interventions based on the action-based model to increase PEB. For this reason, the effect of cognitive dissonance interventions premised on the action-based model to increase PEB should be demonstrated for both the home and work contexts.

*H*1: A cognitive dissonance intervention based on intraindividual information about the interaction of PEA and PEB increases the intention for future PEB (a) in the home context and (b) in the work context.

However, the simple transfer of interventions from the home context to the workplace does not seem possible for several reasons. First, differences in the impact of interventions can be assumed due to the fact that behavior at the workplace is to a large extent not self-determined (Littleford et al., 2014). Furthermore, work behavior does not primarily serve the achievement of climate goals, but is rather geared towards the achievement of economic goals (Kotlar et al., 2018). Accordingly, it can be assumed that the interaction of PEB and PEA in the sense of the action-based model increases a state of arousal in both the home and the work context. However, in the work context, this state of arousal cannot be reduced by a behavioral change due to the restricted scope for action (Littleford et al., 2014). In particular, the need for autonomy to perform PEB has not been sufficiently investigated in past studies (Norton et al., 2015) and could be a key factor for increasing PEB in the work context (Yuriev et al., 2018), which needs to be considered regardless of the intervention type. Based on these findings, it is reasonable to assume that interaction effects differ between the two contexts.

*H*2: The effects of cognitive dissonance intervention based on individual information about the interaction of PEA and PEB on the intention for future PEB are higher in the home than in the work context.

For this reason, the situational conditions of both contexts must be diagnosed and taken into account so that interventions to increase PEA and PEB can be successfully implemented in both the home and work context (Bamberg and Möser, 2007; McDonald, 2014). Studies demonstrate that the contextual conditions for showing pro-environmental behavior differ significantly between home and work contexts. In particular, the scope of action differs between home and work contexts. The lack of autonomy seems to be one of the main obstacles in the work context that hinders PEB (Yuriev et al., 2018). The differences diagnosed in H2 between the home and work contexts may be due attributable to the autonomy available to PEB.

*H*3: The level of autonomy to show PEB in the home sector is significant higher compared to the work context.

The application of the action-based model, especially in the work context, can only be successful if the existing model, in addition to the intraindividual interaction of PEA and PEB, is extended by situational factors that may inhibit the effect of the cognitive dissonance intervention. Thus, the interaction from person and context provides additional information about the effect and future implementation of methods to increase PEB. As described above, consideration of autonomy for PEB appears to influence the effect of the cognitive dissonance manipulation. To examine whether autonomy actually has an impact on the effect of the cognitive dissonance intervention, according to the action-based model, there must be a three-way interaction of current PEA and PEB, as well as the amount of autonomy.

*H*4: The three-way interaction of PEA, PEB and autonomy for PEB increases the behavioral intention for future PEB. The level of autonomy significantly increases PEB.

**Figure 2:**
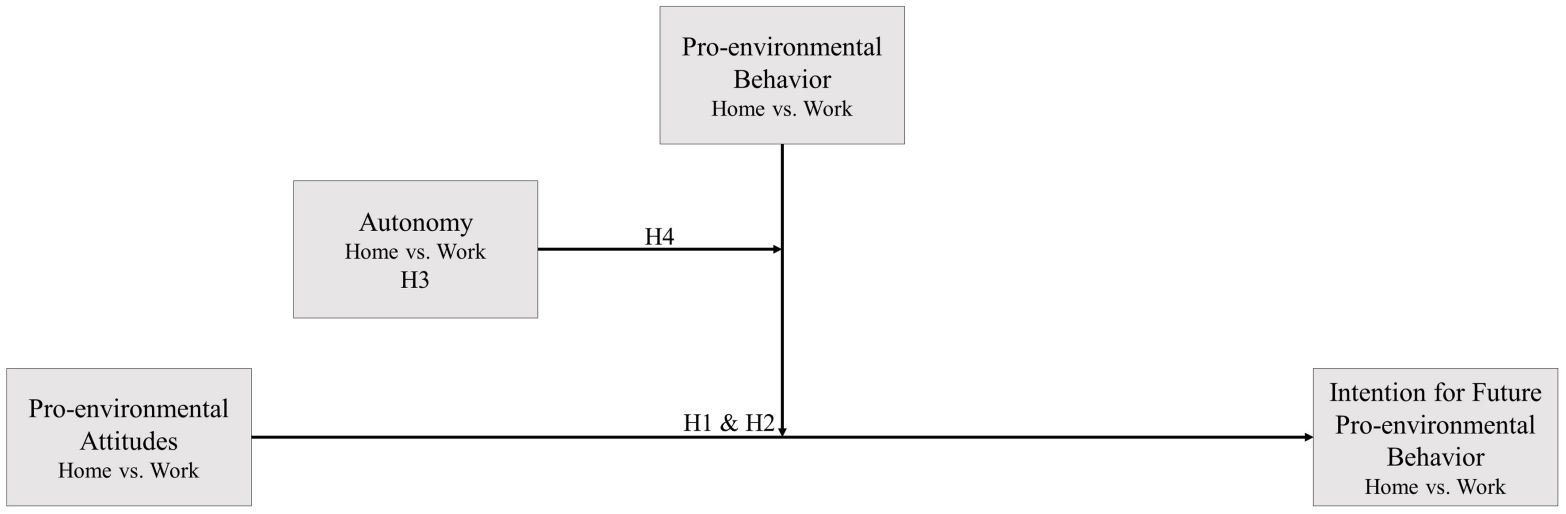
Graphical overview of the assumed hypotheses.

For a better overview of these assumptions, the relationships between the variables assumed in the hypotheses are visually illustrated in Figure 2.

## 3. Methods

### 3.1. Sample

Participants were recruited online via postings on social networks or by contacting participants directly on these platforms. Participation in the study was voluntary and not compensated. Based on estimated mean effect sizes, the sample size was calculated to 162 using the tool G-Power (Faul et al., 2009). A total of *N* = 152 German individuals aged 18–59 years (*M* = 28.93, *SD* = 10.91) participated in the study. Only complete data sets of the participants were considered for the analysis of the study. Participants with missing data were excluded from the analysis. 59.8% reported their gender as female, 39.5% as male, and 0.7% as diverse. The majority of participants reported being fulltime employed (71.7%) and the remaining participants (28.3%) were still in education at the time of study completion. The most common educational qualification in the sample was the general qualification for university entrance (27.6%), followed by a completed apprenticeship (25.7%) and a university degree (20.4%). 11.8% of the participants reported a secondary school certificate as their highest educational qualification, 11.2% completed a technical college entrance qualification and 2.6% completed lower secondary school. Compared to the entire German population, this sample can be considered representative of the population. The age range of the participants represents almost the entire working age. The level of education is also comparable with the German population as a whole (Statistisches Bundesamt, 2020). Only the gender distribution does not correspond to the German population. However, since no gender effects are assumed in this study, this should not result in any bias in the results. Mahalanobis distances were calculated to diagnose possible outliers in the questionnaire instruments used (Daszykowski et al., 2007). At the value of *p* level of 0.001, no outliers were found in this sample.

### 3.2. Design

To test the hypotheses, we used a study design similar to that used in aptitude-treatment interaction studies (McLeod et al., 1978; Astleitner and Koller, 2006), in which we randomly assigned participants to a home-context or a work-context condition. However, participants without work experience were automatically assigned to the home context condition. Half of the participants were asked to complete the questionnaire concerning their PEA and PEB for the home context, and the other half of the sample was asked to provide the information for the work context. The aptitude-treatment design allows to test the influence of situational factors on individual psychological constructs. In the present study, it can be assumed that the interaction of PEA and PEB leads to different expressions of the interaction effect depending on the context. To measure the effect of a cognitive dissonance intervention on future PEB intentions, we induced intraindividual cognitive dissonance via feedback on the amount of individual PEB and PEA scores of each participant. The present study was reviewed by the Ethics Committee of Bielefeld University and found to be ethically unobjectionable.

#### 3.2.1. Cognitive dissonance intervention

According to the action-based model, to create intraindividual cognitive dissonance each participant received a report based on their individual PEA and PEB. This procedure fed back to participants as to whether their real PEA and PEB were congruently expressed or contrary with each other.

Participants were fed back whether their PEA and PEB were below average or above average. Feedback could be given according to the following four expressions (Table 1).

**Table 1:** Feedback variations of intraindividual pro-environmental attitudes and pro-environmental behavior expressions.

	PEA below average	PEA above average
PEB below average		
PEB above average		

In accordance with the action-based model, this approach should produce cognitive dissonance for participants with contrarily expressed PEA and PEB. The participants with balanced expressions of PEA and PEB should not experience an arousal state.

#### 3.2.2. Procedure

The study was conducted online with the survey tool Qualtrics, which was used to allow participants to complete the classic questionnaire instruments regardless of location and time. Furthermore, Qualtrics offers the possibility to evaluate the data of the participants in real time and to give appropriate feedback on this evaluation. This function was used in particular for the creation of the cognitive dissonance. After participants gave informed consent at the beginning of the study, they were asked to provide their sociodemographic data. This was followed by the randomized context assignments of the participants to the work context or the home sphere items. As the first questionnaire measure, participants were asked to rate their individual and context specific PEA and PEB via self-report, after which the available autonomy for PEB was assessed. Following the self-reported feedback of individual PEA and PEB, a manipulation check was performed by checking the participants’ arousal state. In the last questionnaire section, participants were asked to report their intention for future PEB in the self-report. Finally, participants had further opportunity to refuse the use of their data before being informed about the purpose of the study.

### 3.3. Measures

In the present study, four constructs were measured for testing the hypotheses: Pro-environmental attitudes, pro-environmental behavior, autonomy and the intention for future pro-environmental behavior. Table 2 shows detailed information of the measures used in this study.

**Table 2:** Overview of the measures used in this study.

Construct	Source	Sample item	Scaling
PEA	Bentler and Maier (2016)	For efficient use of light, motion detectors should be installed in my house/company building. The first choice of transport for private travel/business trips should always be the train. My household/my employer should only purchase recycled paper for the printer. When purchasing a printer for my household/work activity, care should be taken to ensure that it prints particularly economically. When purchasing new furniture for my household/work, care should be taken to ensure that it is sustainably produced. I would support the introduction of rules for ecological behavior in my household/organization.	7-point Likert scale from 1 “strongly disagree” to 7 “strongly agree”
PEB			
Current PEB	Küstner (2020)	How often do you eat meat in a private context/during work? Which description most closely fits the foods you eat in the private context/during work? How many square meters do you personally have available in the private context/during work and what is your heating behavior? How regularly do you use a car in the private context/work context? How often have you traveled by plane in the past few years in a private/work context? How would you most likely rate your consumption behavior/the consumption behavior of your employer?	5-point Likert scale with alternating naming of scale ends
IfFPEB	Bentler and Maier (2016)	I make sure to use appliances with high energy efficiency ratings in the private/work context. I try to plan trips/work trips so that I can combine several occasions/tasks in nearby places on one way. In everyday life/During work, I try to eat regional and seasonal products. When shopping groceries for my household/organization, I make sure to avoid trash. I talk to my family and friends/colleagues about ecological behavior. In my household/During work, I separate my waste correctly and use the bins provided for this purpose.	7-point Likert scale from 1 “strongly disagree” to 7 “strongly agree”
AfPEB	Hackman and Oldham (1974); Schmidt (2010)	In terms of sustainability, how much autonomy do you have in your home/during your work? That is, to what extent can you determine how environmentally conscious you behave at home/during work? There is considerable opportunity in the home/during work to decide for myself how much I want to exhibit sustainable behavior. I have no opportunity at all to take personal initiative and autonomy in implementing environmentally conscious behavior at home/during work.	7-point Likert scale from 1 “totally incorrect” to 7 “totally correct”
CD	Priolo et al. (2016)	Please indicate whether you agree or disagree with the following statements. I am feeling… Tense Tangled Embarrassed Uncomfortable	7-point Likert scale from 1 “do not agree at all” to 7 “agree completely”

PEA, Pro-environmental Attitudes; PEB, Pro-environmental Behavior; IfFPEB, Intention for Future PEB; AfPEB, Autonomy for PEB; CD, Cognitive Dissonance.

To check the independence of the instruments, a confirmatory factor analysis was calculated. For this purpose, in addition to the comprehensive model, submodels were also calculated using only the data for the home and work contexts. As can be seen from Table 3, the four independent factor model shows a good model fit in all cases, which is significantly better than the single factor model.

**Table 3:** Results of confirmatory factor analysis of the model.

Model	*χ* ^2^	*df*	*χ*^2^/*df*	NFI	CFI	RMSEA
Model 1: Four factors, overall	354.21	183	1.94	0.78	0.88	0.08
Model 1: Four factors, home sphere	244.67	183	1.34	0.75	0.92	0.07
Model 1: Four factors, work sphere	306.74	183	1.68	0.65	0.82	0.09
Model 2: One factor	681.50	189	3.61	0.58	0.65	0.13

Table 4 also provides detailed information on item loadings, average variance explained, composite reliability, and Cronbach’s alpha. The data shows satisfactory to good values. Only the values of the Autonomy for PEB in the work context are insufficient.

**Table 4:** Detailed results of item loadings, average variance explained, composite reliability, and Cronbach’s alpha.

		Overall						Home sphere						Work sphere						*β*	*SE*	CR	AVE	C. Rel.	*α*	*β*	*SE*	CR	AVE	C. Rel.	*α*	*β*	*SE*	CR	AVE	C. Rel.	*α*
PEA	PEA1	0.52	0.09	6.38	0.50	0.81	0.85	0.54	0.13	4.74	0.52	0.87	0.86	0.50	0.13	4.35	0.49	0.85	0.85
	PEA2	0.66	0.10	8.43				0.63	0.14	5.73				0.68	0.15	6.13			
	PEA3	0.76	0.10	9.99				0.76	0.13	7.50				0.75	0.14	6.81			
	PEA4	0.77	0.89	10.12				0.79	0.12	7.83				0.77	0.12	6.97			
	PEA5	0.71	0.86	9.16				0.73	0.14	7.05				0.69	0.13	6.23			
	PEA6	0.81						0.85						0.77					
PEB	PEB1	0.69	0.15	7.44	0.41	0.81	0.80	0.79	0.16	7.32	0.51	0.86	0.85	0.52	0.26	3.74	0.32	0.74	0.74
	PEB2	0.66	0.12	7.13				0.75	0.12	6.82				0.57	0.22	4.00			
	PEB3	0.58	0.15	6.39				0.61	0.16	5.34				0.47	0.25	3.45			
	PEB4	0.64	0.13	7.00				0.70	0.13	6.30				0.60	0.24	4.19			
	PEB5	0.54	0.16	5.95				0.58	0.17	5.05				0.62	0.20	4.29			
	PEB6	0.71						0.83						0.59					
IfFPEB	IfFPEB1	0.68	0.22	6.15	0.49	0.86	0.84	0.63	0.22	4.79	0.51	0.86	0.86	0.69	0.48	3.62	0.49	0.85	0.84
	IfFPEB2	0.67	0.21	6.09				0.70	0.21	5.25				0.61	0.42	3.46			
	IfFPEB3	0.83	0.23	6.84				0.77	0.20	5.65				0.89	0.57	3.93			
	IfFPEB4	0.76	0.22	6.54				0.77	0.21	5.66				0.73	0.47	3.72			
	IfFPEB5	0.70	0.25	6.27				0.74	0.26	5.49				0.75	0.56	3.75			
	IfFPEB6	0.55						0.66						0.44					
AfPEB	A1	0.72	0.12	8.02	0.58	0.81	0.81	0.93	0.31	5.63	0.57	0.80	0.77	0.26	0.23	2.10	0.42	0.64	0.62
	A2	0.78	0.11	8.41				0.65	0.21	4.82				0.96	0.54	2.89			
	A3	0.79						0.66						0.53					

Home sphere *n*, 72; Work sphere *n*, 80; Overall *N*, 152; PEA, Pro-environmental attitudes; PEB, Pro-environmental behavior; IfFPEB, Intention for future PEB; AfPEB, Autonomy for PEB; *β*, standardized regression coefficient, SE, standard error; CR, critical ratio; AVE, average variance extracted; C. Rel., composite reliability; *α*, Cronbach’s alpha/internal consistency.

#### 3.3.1. Pro-environmental attitudes

To measure PEA, the environmentally friendly attitudes and behavior in the workplace questionnaire (Bentler and Maier, 2016) was used in the present study. The participants were asked to answer six items for self-assessment of their PEA. One item each of the questionnaire refers to attitudes towards recycling, ecological consumption, waste reduction, proactive environmental behavior, energy saving and mobility. Since the original questionnaire was developed for the work context, the item texts for the home sphere condition in this study were adapted to this context. Participants answered on a seven-point Likert scale ranging from 1 “strongly disagree” to 7 “strongly agree.”

#### 3.3.2. Pro-environmental behavior

##### 3.3.2.1. Current pro-environmental behavior

Pro-environmental behavior was surveyed via two different instruments in this study. As an independent variable, PEB was assessed via the ecological footprint by participants in a self-report (Küstner, 2020). A total of six items were used to determine the consumption of natural resources for the home or work sphere. Since the original version of the scale does not ask about ecological lifestyle in a context-specific way, the participants in the present study were asked via the instruction to evaluate their own behavior only for the home or work context. The content of the questionnaire items related to the factors dietary habits, heating behavior, car use, long-distance flights and consumption habits. The answers were given on a five-point scale ranging from 1 to 5. In contrast to all other scales used in this study, a value of 1 in this scale represents high expression of PEB, whereas five represents low expression of PEB.

##### 3.3.2.2. Intention for future pro-environmental behavior

The scale for measuring environmentally friendly attitudes and behavior in the work context (Bentler and Maier, 2016) was used as the dependent variable for measuring the intention for future PEB. A total of six items required participants to indicate how likely they were to perform the behavior being assessed in the future. The behaviors described in the scale relate to recycling, ecological consumption, waste reduction, proactive environmental behavior, energy saving and mobility. Since the scale was originally designed only for the work context, the item texts for the home sphere condition in this study were adapted to this context. To answer the questionnaire items, participants were provided with a seven-point Likert scale ranging from 1 “strongly disagree” to 7 “strongly agree.”

#### 3.3.3. Autonomy for pro-environmental behavior

To measure autonomy for PEB, a German version of the Job Diagnostic Survey was used (Hackman and Oldham, 1974; Schmidt, 2010). The questionnaire was originally designed to measure general autonomy within work activities. In the present study, participants were asked to rate only the autonomy for their PEB in a context-specific manner for the work or home context. The scale includes three items that had to be rated on a seven-point Likert scale ranging from 1 low extent of autonomy for PEB to 7 high extent of autonomy for PEB.

#### 3.3.4. Cognitive dissonance

To measure the participants’ state of arousal as a manipulation check, the cognitive dissonance thermometer (Priolo et al., 2016) was used. The participants had to rate their current state of arousal via this instrument by rating four adjectives, e.g., tense and uncomfortable. For this purpose, they were provided with a seven-point Likert scale ranging from 1 “do not agree at all” to 7 “agree completely.” With a value of α 0.90, the internal consistency was in the good range.

## 4. Results

Table 5 presents the descriptive statistics of the used constructs. The correlations correspond to the intuitive assumptions. It should be noted that the ecological footprint scale to measure PEB is inverted. A low scale value represents a high level of PEB and a high scale value represents a low expression of PEB.

**Table 5:** Descriptive statistics and correlations of the constructs used in this study.

	Home sphere		Work sphere		PEA	PEB	IfFPEB	AfPEB
	*M*	*SD*	*M*	*SD*				
PEA	4.91	1.27	5.07	1.30				
PEB	2.77	0.86	2.35	0.74	−0.60*			
IfFPEB	5.16	1.24	4.89	1.40	0.72*	−0.51*		
AfPEB	5.32	1.17	3.43	1.26	0.22*	−0.02	0.41*	
CD	3.22	1.64	3.11	1.64	0.29*	0.08	0.35*	0.03

Home sphere *N* = 72, Work sphere *N* = 80; ^*^The correlation is significant at the level of *p* 0.01; PEA, pro-environmental attitudes; PEB, pro-environmental behavior; IfFPEB, intention for future PEB; AfPEB, autonomy for PEB; CD, cognitive dissonance.

To check the manipulation of cognitive dissonance in this study, the interaction effect of PEA and PEB on the dissonance thermometer using Model 1 of the PROCESS macro for SPSS with 5,000 bootstrap repetitions (Hayes, 2018) was computed. According to the action-based model the interaction of PEA and PEB increases cognitive dissonance significantly, *R^2^* = 0.46, Δ*R^2^* = 0.03, *F* (1, 147) = 6.24, *p* 0.014, 95% CI [0.06, 0.52], which means that the intervention on the basis of the feedback of the individual expression of PEA and PEB works to establish an arousal state.

To test Hypothesis 1, according to the action-based model, we computed an interaction of participants’ PEA and PEB using Model 1 of the PROCESS macro for SPSS with 5,000 bootstrap repetitions (Hayes, 2018). As shown in Table 6, the interaction from PEA and PEB significantly affected the intention for future PEB, *R^2^* = 0.56, Δ*R^2^* = 0.03, *F* (1, 148) = 10.72, *p* 0.001, 95% CI [0.09, 0.37].

**Table 6:** Interaction between pro-environmental attitudes and pro-environmental behavior on intention for future pro-environmental behavior.

	Estimate	*SE*	95% CI		*p*
			LL	UL	
Constant	6.36	1.39	3.62	9.11	0.00
PEA	−0.07	0.24	−0.54	0.39	0.75
PEB	−1.47	0.40	−2.27	−0.68	0.00
PEA x PEB	0.23	0.07	0.09	0.37	0.00

PEA, pro-environmental attitudes; PEB, pro-environmental behavior.

**Figure 3:**
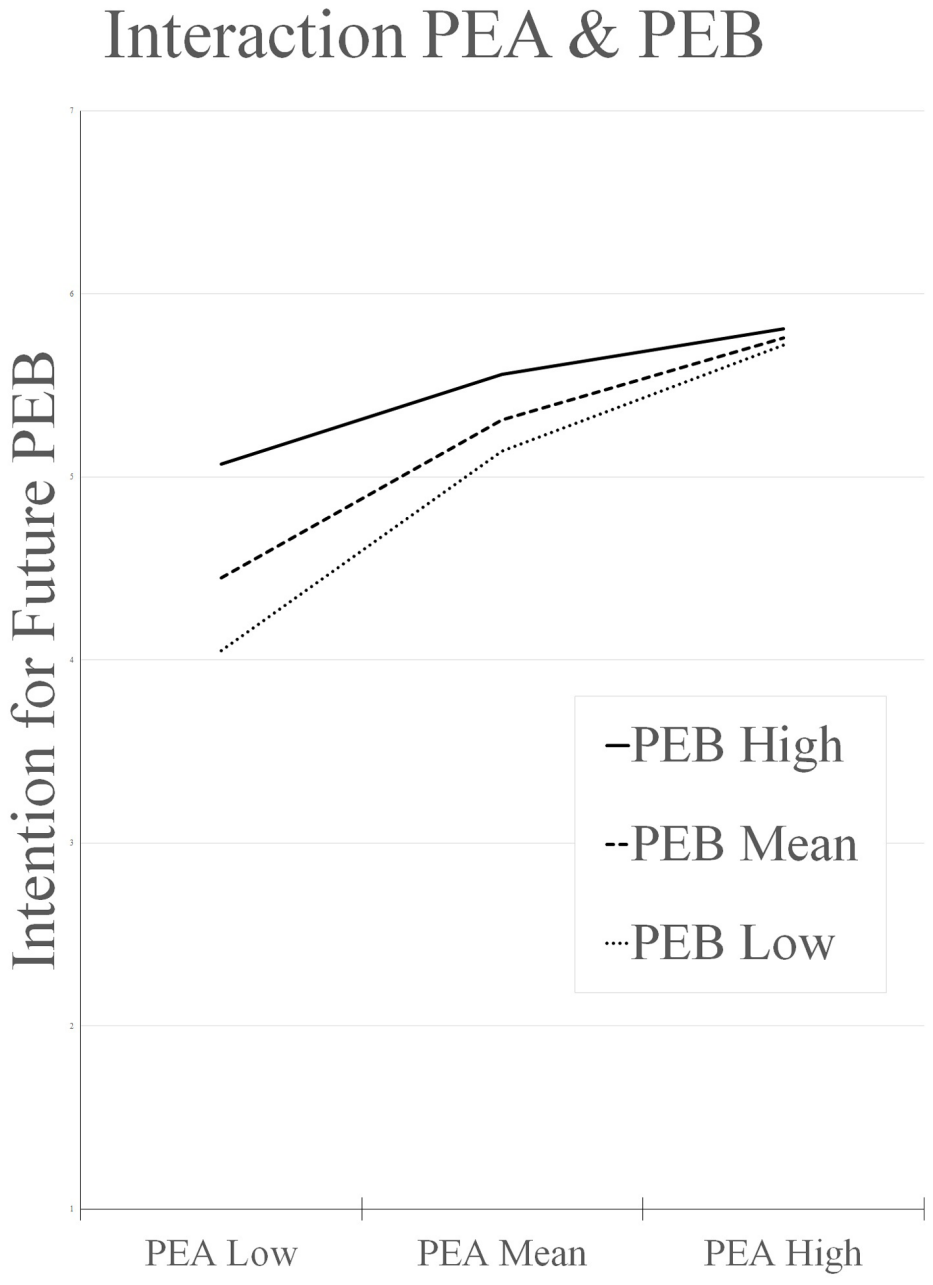
Conditional pro-environmental attitudes and pro-environmental behavior interaction on intention for future pro-environmental behavior. PEA, pro-environmental attitudes; PEB, pro-environmental behavior.

As illustrated in Figure 3, the interaction of PEA and PEB especially leads to different expressions of intention for future PEB when individual PEA is low. If the individual expression of PEA is low and the current PEB is high, this leads to a significant increase in intention future PEB; see also Table 7. Hypothesis 1 is confirmed.

**Table 7:** Conditional effects of pro-environmental attitudes at values of pro-environmental behavior on intention for future pro-environmental behavior.

PEA	PEB	IfFPEB *M*
Low	Low	4.05
Mean	Low	5.14
High	Low	5.72
Low	Mean	4.45
Mean	Mean	5.31
High	Mean	5.76
Low	High	5.07
Mean	High	5.56
High	High	5.81

PEA, pro-environmental attitudes; PEB, pro-environmental behavior; IfFPEB, intention for future PEB.

Concerning hypothesis 2, similar to hypothesis 1, we computed an interaction of participants’ PEA and PEB using Model 1 of the PROCESS macro for SPSS with 5,000 bootstrap repetitions, but split the study data separately for the home and work spheres. Significant interactions between PEA and PEB were found for both the home sphere (*R*^2^ = 0.66, Δ*R*^2^ = 0.04, *F* (1, 72) = 43.65, *p* 0.000, 95% CI [0.08, 0.47]) and the work context (*R*^2^ = 0.56, Δ*R*^2^ = 0.03, *F* (1, 80) = 32.17, *p* 0.000, 95% CI [0.03, 0.41]). As shown in Table 8, the interaction effects from PEA and PEB on intention for future PEB are higher in the home than in the work sphere. That means that hypothesis 2 is confirmed. Which situational factors are responsible for these differences in interaction effects will be examined in the following.

**Table 8:** Interaction between pro-environmental attitudes and pro-environmental behavior on intention for future pro-environmental behavior separated for the home and the work sphere.

	Estimate	*SE*	95% CI		*p*
			LL	UL	
Home sphere
Constant	7.48	2.14	3.22	11.74	0.00
PEA	−0.23	0.35	−0.94	0.48	0.52
PEB	−1.71	0.60	−2.91	−0.52	0.01
PEA × PEB	0.27	0.10	0.08	0.47	0.01
Work sphere
Constant	7.01	1.79	3.44	10.57	0.00
PEA	−0.12	0.31	−0.74	0.50	0.70
PEB	−1.72	0.54	−2.79	−0.64	0.00
PEA × PEB	0.22	0.10	0.03	0.42	0.02

PEA, pro-environmental attitudes; PEB, pro-environmental behavior.

According to hypothesis 3, there should be significantly more autonomy to show PEB in the home context than in the work context. To test this hypothesis, a t-test for independent samples was calculated. In the home context (*M* = 5.32, *SD* = 1.17), the level of autonomy to show PEB was significantly higher than in the work context (*M* = 3.43, *SD* = 1.16), *t* (150) = 9.54, *p* 0.000, *d* 1.22, 95% CI [1.18, 1.91]. Hypothesis 3 is confirmed.

To test the fourth hypothesis, a three-way interaction of PEA, PEB as well as autonomy for PEB was calculated. The aim was to test whether autonomy adds an incremental contribution to the intention for future PEB in addition to the interaction from PEA and PEB. To test this hypothesis, we used Model 3 of the PROCESS macro for SPSS with 5,000 bootstrap repetitions (Hayes, 2018). As shown in Table 9, the three-way interaction of PEA, PEB and autonomy significantly affects the intention for future PEB, *R*^2^ = 0.66, Δ*R*^2^ = 0.01, *F* (1, 144) = 4.89, *p* 0.029, 95% CI [0.01, 0.17].

**Table 9:** Three-way interaction between pro-environmental attitudes, pro-environmental behavior and autonomy for pro-environmental behavior on intention for future pro-environmental behavior.

	Estimate	*SE*	95% CI		*p*
			LL	UL	
Constant	−0.43	3.72	−7.78	6.91	0.91
PEA	1.18	0.63	−0.06	2.43	0.06
PEB	0.16	0.98	−1.78	2.10	0.87
PEA × PEB	−0.17	0.17	−0.51	0.17	0.33
AfPEB	1.80	0.92	−0.01	3.62	0.05
PEA × AfPEB	−0.32	0.15	−0.62	−0.02	0.04
PEB x AfPEB	−0.41	0.25	−0.90	0.07	0.10
PEA × PEB × AfPEB	0.09	0.04	0.01	0.17	0.03

PEA = pro-environmental attitudes; PEB, pro-environmental behavior; AfPEB, autonomy for pro-environmental behavior.

The intention for future PEB increased significantly due to the amount of autonomy for PEB; see Table 10. The more autonomy available, the higher the intention for future PEB.

**Table 10:** Conditional effects at values of pro-environmental attitudes, pro-environmental behavior and autonomy for pro-environmental behavior on intention for future pro-environmental behavior.

AfPEB	PEA	PEB	IfFPEB *M*
Low	Low	Low	3.46
Low	Mean	Low	4.40
Low	High	Low	4.90
Low	Low	Mean	3.89
Low	Mean	Mean	4.76
Low	High	Mean	5.21
Low	Low	High	4.55
Low	Mean	High	5.29
Low	High	High	5.68
Mean	Low	Low	4.21
Mean	Mean	Low	5.11
Mean	High	Low	5.58
Mean	Low	Mean	4.74
Mean	Mean	Mean	5.36
Mean	High	Mean	5.69
Mean	Low	High	5.53
Mean	Mean	High	5.74
Mean	High	High	5.85
High	Low	Low	4.71
High	Mean	Low	5.59
High	High	Low	6.04
High	Low	Mean	5.30
High	Mean	Mean	5.77
High	High	Mean	6.02
High	Low	High	6.19
High	Mean	High	6.05
High	High	High	5.97

PEA, pro-environmental attitudes; PEB, pro-environmental behavior; AfPEB, Autonomy for PEB; IfFPEB, intention for future PEB.

**Figure 4:**
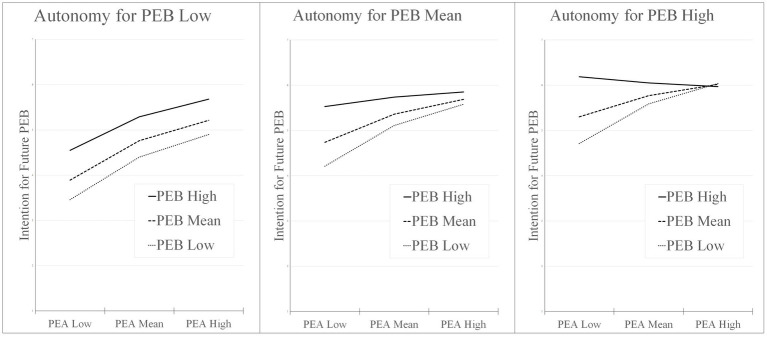
Conditional pro-environmental attitudes and pro-environmental behavior interaction at low, mean and high values of autonomy for pro-environmental behavior on intention for future pro-environmental behavior. PEA, pro-environmental attitudes; PEB, pro-environmental behavior.

As can be seen in Figure 4, the effect strength increases depending on the degree of autonomy. The more autonomy available for PEB, the stronger the interaction effect. The effect of cognitive dissonance on increasing future PEB was strongest when individuals had a low level of PEA, a high level of PEB and a high level of autonomy for PEB. These results confirm hypothesis 4.

## 5. Discussion

### 5.1. General discussion

In the present study, we explored the theoretical assumption of the action-based model for creating cognitive dissonance in the field of environmental sustainability and how cognitive dissonance can be used to increase PEB intentions in the home and work contexts. First, the results of our study show that intraindividual feedback on individual PEA and PEB can be used to create cognitive dissonance. This cognitive dissonance significantly increases the intention for future PEB. The interaction of PEA and PEB has a significant positive effect on the intention for future environmentally friendly behavior if the extent of individual PEA and PEB are unevenly expressed. Second, our analysis revealed that situational circumstances influence the extent of PEB by examining the autonomy to show PEB. There is significantly more autonomy for PEB in the home context than in the work sphere. Third, we were able to demonstrate the importance of autonomy for PEB via the three-way interaction of attitudes, behavior and autonomy. Autonomy for PEB additionally increases intention for future PEB. Based on the theoretical foundation of the action-based model, we were thus able to validate the application of cognitive dissonance in the application domain of PEB as well as additionally identify situational constructs that influence PEB intentions.

### 5.2. Theoretical implications

The present work is the only study to date in the field of psychological environmental sustainability research that has been able to confirm the use of the action-based model (Harmon-Jones et al., 2015) to create cognitive dissonance as well as to increase sustainable behavioral intentions. Accordingly, the perceived inconsistency between PEA and PEB leads to an arousal that is subsequently reduced by the intention for a behavioral change in which PEB is increased. To establish cognitive dissonance, participants’ real attitude and behavior scores were fed back. Even though the attitudinal and behavioral data were based on subjective self-assessments of the participants, the study can be considered internally valid. A major advantage of the present intervention for creating cognitive dissonance is that the perception of intrapersonal variables is sufficient, whereas other procedures for generating cognitive dissonance in the domain of sustainable behavior must rely on additional psychological mechanisms. In the induced hypocrisy method (Priolo et al., 2016; McGrath, 2018), in addition to personal attitudes, the perception of social norms is used to establish an arousal state. Based on the action-based model, this additional information is not needed. The simplicity of the method we used can been seen as an advantage over other methods. Although the action-based model assumes that behavior change is exclusively intraindividually motivated and serves only to lower the state of arousal, future studies should furthermore consider what interindividual mechanisms can be used to additional affect this behavioral change. The study was able to show significant results for the two domains of home and work context. It should be noted that the study focused particularly on office workplaces in the work context due to target group recruitment. Autonomy as a framework condition has a significant influence on the effects of the cognitive dissonance intervention for office workplaces. For other types of workplaces, other framework conditions may well regulate the effects of dissonance methods. It should be noted that environmentally friendly behavior can be evaluated as secondary work behavior, which is only exercised if sufficient resources are available after the completion of the primary work tasks. The complexity of work activities as a framework condition could significantly consume the available resources. Insofar as a work activity is highly complex, employees may no longer have sufficient resources available to complete the activity in an environmentally friendly manner. In addition to complexity, other work design factors could affect environmentally friendly work behavior, which will be considered in future research. A transfer of the results to other areas, for instance tourism activities, should be examined in future studies. When transferring the results to other contexts, situational variables and the interaction of situational and intrapersonal variables should be taken into account. Future studies should diagnose more situational variables that may inhibit or enhance PEB. As demonstrated in the present study, the design of psychological intervention measures can succeed when interactions of intrapersonal and situational variables are considered. In addition, the development of trainings in which people are supposed to reflect on situational conditions in which they have the autonomy to show PEB is another possible research direction. This enables people to work on behavioral changes in the sense of job crafting, especially in the work context.

### 5.3. Practical implications

The described procedure of the intervention for creating cognitive dissonance as well as increasing PEB can be easily applied in practice. The great advantage of the used method is that people can carry out this intervention regardless of time and place. Similar to the established ecological footprint measurement (Wackernagel et al., 1999; Lin et al., 2018), the method used here can be processed online and the individual results can be reported back to the participants. However, while the ecological footprint measurement uses external anchor values for feedback by linking the individual behavior of the participants with the available renewable resources, the advantage in the method we developed is that cognitive dissonance is only generated via the inconsistency of individual attitudes and behavior. Thus, no external influencing factors need to be made salient to generate an arousal state. Although it is likely that there will be a reduction in sustainable behavior, provided that the inconsistency of attitudes and behavior is such that PEA are less distinct than PEB. In our sample, only 2% of participants had a lower expression of PEA than PEB, whereas 33% of participants had higher PEA than PEB, representing a critical amount to achieve an increase in PEB via the method used. Furthermore, the context comparison of this study provides an advantage for transferring the results into practice. We were able to demonstrate that cognitive dissonance leads to an increase in behavioral intention for PEB in both the home sphere and the work context. Accordingly, application of the intervention in these domains is straightforward. In addition, we were able to demonstrate that the autonomy available has a meaningful impact on behavioral change. Accordingly, when transferring our intervention method, care should also be taken to ensure that the target individuals have sufficient autonomy for maneuver to adapt their PEB. For example, employees could decide for themselves whether to travel on business by airplane or railway. It would also be conceivable to increase employees’ right to hear their opinion about the purchase of goods and consumables as well as the company’s energy supply. Nevertheless, it should be noted at this point that the sample of this study was particularly active in office workplaces. The industrial production environment has one of the greatest influences on CO2 pollution (Principles for Responsible Investment, and UNEP Finance Initiative, 2011). However, due to standardized and automated processes of lean management in industrial production, there seems to be little autonomy available for behavioral changes of individuals. In these areas, it makes sense to apply the method presented here as part of organizational development measures through project groups. These project groups can use the measures before designing creative solution strategies of organizational development. Thus, novel processes can be structured in an environmentally friendly way to pursue ecological goals of organizations.

### 5.4. Limitations

Even though the method used to generate cognitive dissonance through feedback of PEA and PEB is a clear advantage of the present study with both scientific added value and high practical transfer, there are also a few points of the present work that show limitations in terms of generalizing the results. From a methodological perspective, it should be noted that the dependent variable is merely an intention to engage in future PEB, which was captured via a self-report. Although previous studies have demonstrated a high correlation between self-reported behavioral measures and actual behaviors exhibited in the area of environmental sustainability (Kormos and Gifford, 2014), future studies should attempt to measure the actual PEB of participants in order to achieve high external validity via an objective measurement procedure.

In the present study, by considering autonomy for PEB, the influence of a situational factor was demonstrated, which can explain differences in the effect of intervention measures between the home and work contexts. In particular, in the work context, behavior is highly regulated by contextual conditions such as the work activity itself, the organizational context, and the expectations of leaders and direct colleagues. For this reason, future studies should investigate other situational conditions that may influence the extent of green behaviors.

### 5.5. Conclusion

The described cognitive dissonance intervention of the present study represents an extremely efficient method to increase the intention to show PEB. The procedure of the intervention by the use of subjective PEA and PEB data can be used both in future psychological experiments and offers beyond that due to the simple application a high transfer into the everyday life. With the consideration of autonomy for PEB, an additional situational factor is taken into account, which has a further starting point on the extent of ecological behavior. The interaction in the present study of individual personal characteristics and situational factors influencing PEB showed promising results and should therefore be considered in future studies. Beyond the scientific value of the cognitive dissonance intervention, the application of this method in everyday life has an added value to contribute the achievement of climate goals like the Paris Climate Agreement.

## Data availability statement

The datasets presented in this study can be found in online repositories. The names of the repository/repositories and accession number(s) can be found at: https://pub.uni-bielefeld.de/record/2977965.

## Ethics statement

The studies involving human participants were reviewed and approved by Bielefeld University. The patients/participants provided their written informed consent to participate in this study.

## Author contributions

DB, GK, and GM contributed to conception and design of the study. GK organized the database. DB performed the statistical analysis and wrote the first draft of the manuscript. All authors contributed to manuscript revision, read, and approved the submitted version.

## Conflict of interest

The authors declare that the research was conducted in the absence of any commercial or financial relationships that could be construed as a potential conflict of interest.

## Publisher’s note

All claims expressed in this article are solely those of the authors and do not necessarily represent those of their affiliated organizations, or those of the publisher, the editors and the reviewers. Any product that may be evaluated in this article, or claim that may be made by its manufacturer, is not guaranteed or endorsed by the publisher.
